# Treatment of children with COVID-19: position paper of the Italian Society of Pediatric Infectious Disease

**DOI:** 10.1186/s13052-020-00900-w

**Published:** 2020-09-24

**Authors:** Elisabetta Venturini, Carlotta Montagnani, Silvia Garazzino, Daniele Donà, Luca Pierantoni, Andrea Lo Vecchio, Giangiacomo Nicolini, Sonia Bianchini, Andrzej Krzysztofiak, Luisa Galli, Alberto Villani, Guido Castelli-Gattinara, for the Italian SITIP-SIP SARS-Cov-2 pediatric infection study group

**Affiliations:** 1 Infection Disease Unit, Meyer Children’s University Hospital, Florence, Italy; 2 Pediatric Infectious Disease Unit, Regina Margherita Children’s Hospital, University of Turin, Turin, Italy; 3 Division of Pediatric Infectious Diseases, Department for Woman and Child Health, University of Padua, Padua, Italy; 4 Pediatric Emergency Unit, Policlinico di Sant’Orsola, Bologna, Italy; 5 Department of Translational Medical Science, Section of Pediatrics, University of Naples Federico II, Naples, Italy; 6 UOC Pediatria, San Martino Hospital, Belluno, Italy; 7 Department of Pediatrics, ASST Santi Paolo e Carlo Hospital, Milan, Italy; 8 Universitarian-Hospital Department Ospedale Bambino Gesù IRCCS, Rome, Italy; 9 Department of Health Sciences, University of Florence, Florence, Italy

**Keywords:** COVID-19, SARS CoV-2, Children, Treatment

## Abstract

A statement of consensus was formulated after reviewing available literature on pediatric treatment strategies for COVID-19 by the Steering and Scientific Committee of the Italian Society of Infectious Pediatric Diseases in connection with the Italian Society of Paediatrics.

## Background

Since December 2019, severe acute respiratory syndrome coronavirus 2 (SARS-CoV-2) infection has been reported in Hubei province, China, and from there it spread worldwide. Starting from the end of February 2020, the number of cases of coronavirus disease 2019 (COVID-19) outside China rapidly increased, urging the World Health Organization (WHO) to declare COVID-19 as a pandemic on March 11 [1].

Most children with SARS-CoV-2 infection develop none or mild symptoms, needing only supportive treatment [2, 3]. However, few cases of severe COVID-19 in children have been reported [3–5], including multisystem inflammatory syndromes [6, 7].

As no evidence of at least good quality is available regarding therapy of COVID-19, treatment regimens of COVID-19 are not standardized. At present, few clinical trials for COVID-19 treatment involving children are ongoing [8]. Moreover, evidences are rapidly evolving and the therapeutic indications can change very quickly and even this work has changed quite a bit in the course of its writing.

To date, many national and international guidelines have been issued for the adult population [9–11]. However, there is a need of guidance on COVID-19 management in children from scientific societies and expert groups. Recently, an expert opinion has been released by a group of pediatric infectious disease specialists from North America, suggesting that supportive care alone is indicated for the overwhelming majority of cases [12].

The Italian Society of Pediatric Infectious Diseases steering and scientific committee developed a position paper on treatment of children with COVID-19, reviewing the current literature on this topic and providing indications based on the available literature data.

Since new evidences will be available in the next weeks/months, the Italian Society of Pediatric Infectious Diseases will guarantee to maintain treatment indication updated on the website www.sitip.org (updated English and Italian versions of the present document).

## Methods

The consensus statement was formulated by the steering and scientific committee of the Italian Society of Pediatric Infectious Diseases in connection with the Italian Society of Pediatrics.

Decision was made after reviewing available literature on pediatric treatment strategies for COVID-19 at 16 June 2020 on Pubmed with the following search strategies: (SARS-CoV-19 OR COVID-19 OR Coronavirus) AND treatment. Moreover, national and international recommendations of WHO and scientific societies available online were evaluated. Ongoing clinical trials were searched on https://clinicaltrials.gov/ and https://www.aifa.gov.it/sperimentazioni-cliniche-covid-19.

### Definitions

Clinical syndromes associated with COVID-19 in children were defined adapting the WHO classification as follows [13–15].

***Asymptomatic case*:** infection identified during screening or contact tracing without symptoms.

***Mild case*:** fever and/or fatigue and/or upper airways symptoms without radiological/ultrasound findings (if performed).

***Moderate case***: fever and/or fatigue and/or upper airways symptoms (cough or mild respiratory distress) and/or poor feeding and/or pneumonia identified with chest X-ray or ultrasound.

***Severe case***:

- Fever and cough, plus at least one of the following:
- Oxygen saturation on finger pulse (SpO2) < 92% on room air
- Severe respiratory distress (grunting, severe chest indrawing), cyanosis, intermittent apnea
- Fast breathing (regardless of fever and crying): respiratory rate (RR) in breaths/minute > 60 < 3 months; > 50 3–12 months; > 40 1–5 years; > 30 > 5 years)
- Systemic symptoms: drowsiness, lethargy, seizures, dehydration

***Critical case*:**

- Pediatric acute respiratory distress syndrome (PARDS)
- Sepsis-associated organ dysfunction
- Septic shock
- Coma

PARDS was defined according to Pediatric Acute Lung Injury Consensus Conference Group definition [16].

Sepsis-associated organ dysfunction and septic shock were defined according to Surviving Sepsis Campaign definition [17].

Regardless of the early stage of the disease, the following indicators should be assessed as related to an increased risk of rapid progression to the severe/critical stage.

***Clinical early warning indicators*:**

- Increased tachypnoea, despite 2 h of intravenous rehydration and low flow nasal cannula oxygen therapy
- Impaired consciousness
- Progressive increasing of lactate values
- Bilateral lung infiltration or multiple lobes involvement, pleural effusion or rapid progression of the lesions in a short period of time
- Age < 3 months
- Underlying diseases (congenital heart disease, bronchopulmonary dysplasia, anomalies of respiratory tract, abnormal hemoglobin, anemia, severe malnutrition, congenital or acquired immunodeficiency)

#### Multisystem inflammatory syndrome

Children and adolescents 0–19 years of age with fever > 3 days AND two of the following:

- Skin rash or bilateral non-purulent conjunctivitis or muco-cutaneous inflammation signs (oral, hands or feet)
- Hypotension or shock
- Signs of myocardial dysfunction, pericarditis, valvulitis, or coronary abnormalities (including echocardiography findings or elevated Troponin/NT-proBNP)
- Evidence of coagulopathy
- Acute gastrointestinal problems (diarrhea, vomiting, or abdominal pain)

AND

Elevated markers of inflammation such as erythrocyte sedimentation rate, C-reactive protein, or procalcitonin.

AND

No other obvious microbial cause of inflammation, including bacterial sepsis, staphylococcal or streptococcal shock syndromes.

AND

Evidence of COVID-19 (positive real-time polymerase chain reaction, antigen test or serology), or a likely contact with patients with COVID-19.

#### Treatment approach based on the different scenarios

***Asymptomatic cases:*** *no treatment.*

***Moderate and mild cases*:** only antipyretic therapy.

***Severe and critical cases****:*

**Table:** 

**Remdesivir** if not available **Hydroxychloroquine OR Lopinavir/ritonavir**	

all these drugs should be preferably administered within a clinical trial.

**Warning:** Hydroxychloroquine can cause QTc prolongation. Every 2 days perform electrocardiogram (ECG) and QTc assessment for QTc prolongation increased risk. For hydroxychloroquine, carry out glucose 6 phosphate dehydrogenase (G6PDH) dosage in case of risk factors for deficiency.

Remdesivir should be administered in patients with normal renal function. Liver function assessment should be performed in all patients prior to starting remdesivir and every other day while receiving remdesivir.

**Duration of therapy**: 5–7 days, extendable according to clinical course.

**Immunomodulating therapy:**

This therapy must be considered in case of:

- ARDS or progressive deterioration of respiratory function.

- Multisystem inflammatory syndrome.

- Marked alteration or increasing trend of IL-6 and/or D-dimer and/or ferritin and/or C-reactive protein.

- Interval of at least 7 days from the beginning of the symptoms.

**Table:** 

**Methylprednisolone or dexamethasone** OR **Anakinra (or Tocilizumab)**	

The available dosages and formulations of the various drugs are indicated in the text. COVID-19 management and treatment in children, according to disease severity, are reported in Table 1.

**Table 1:** COVID-19 management and treatment in children, according to disease severity

Clinical picture	Supportive care	Antiviral treatment
**Asymptomatic infection**	None	None
**Mild case:** fever and/or asthenia with upper respiratory signs	None In case of fever > 38 °C: paracetamol	None
**Moderate case:** fever and/or asthenia and/or respiratory signs/symptoms, such as cough, mild distress with polypnea and/or difficulty in feeding, signs of dehydration	• Airway suction in case of obstruction • Oxygen therapy using nasal cannulas or facial mask with Venturi system (if oxygen saturation in air < 95%) • Intravenous access, adequate fluid and caloric intake based on hydration status • Give paracetamol in case of fever > 38 °C • Monitor vital signs (Bedside-PEWS )[18] every 8 h (or before in case of changes in the clinical picture)	None
**Severe illness:** - SpO2 < 92% on finger pulse oximeter taken at rest - Labored breathing (moaning, nasal flattering, sternal, clavicular and internal recesses ribs), cyanosis, intermittent apnea - Tachypnea, in apyrexia and absence of crying (respiratory rate > 60 breaths/minute < 3 months; > 50 breaths /minute 3–12 months; > 40 breaths /minute 1–5 years; > 30 breaths/ minute > 5 years) - Systemic signs of worsening: lethargy, inability to feed/drink, convulsions - Suspected sepsis - Shock or other organ failure requiring care	• Airway suction in case of obstruction • Oxygen therapy using nasal cannulas or facial mask with Venturi system or High Flow Nasal Cannula or Non-Invasive ventilation (target oxygen saturation > 95%), refer to WHO Interim guidance • Intravenous access, adequate fluid and caloric intake based on hydration status. Monitor urinary output. • Give paracetamol in case of fever > 38 °C • Monitor vital signs (Bedside-PEWS )[18] every 8 h (or before in case of changes in the clinical picture) • Avoid empiric antibiotic treatment if no evidence of bacterial infection (consult an infectious disease specialist or refer to hospital guidelines) • Consider immunomodulation: methylprednisolone or interleukin inhibitors if available (Anakinra or Tocilizumab) • Consider venous thromboembolism prevention: low molecular-weight heparin	Remdesivir if not available Hydroxychloroquine OR Lopinavir/ritonavir
**Critical illness** ARDS Sepsis-associated organ dysfunction Septic shock Coma	• Airway suction in case of obstruction • Oxygen therapy using nasal cannulas or facial mask with Venturi system or High Flow Nasal Cannula or Non-Invasive Ventilation (target oxygen saturation > 95%). In case of mechanical ventilation refer to WHO Interim guidance • Intravenous access, adequate fluid and caloric intake based on hydration status. Monitor urinary output. • Give paracetamol in case of fever > 38 °C • Monitor vital signs (Bedside-PEWS )[18] every 8 h (or before in case of changes in the clinical picture) • Avoid empiric antibiotic treatment if no evidence of bacterial infection (consult an infectious disease specialist or refer to hospital guidelines) • Add immunomodulation: methylprednisolone or interleukin inhibitors if available (Anakinra or Tocilizumab) • Add venous thromboembolism prevention: low molecular-weight heparin	Remdesivir if not available Hydroxychloroquine OR Lopinavir/ritonavir

#### Supportive care

**Antipyretic therapy:** prefer paracetamol (10–15 mg/kg every 4–6 h) in case of fever > 38.5 °C. Avoid ibuprofen in case of dehydration, vomiting and diarrhea, as it is associated with an increased risk of kidney failure. Some authors have suggested a correlation between the use of ibuprofen and an unfavorable course of SARS-Cov-2 infection [19]. However, these data are not currently confirmed and the European Medicines Agency does not contraindicate the use of non-steroidal anti-inflammatory drugs [20].

**Inhalation therapy:** if topic steroids and/or bronchodilators are needed (e.g. patient with recurrent wheezing undergoing exacerbation and suggestive symptoms or confirmed SARS-Cov-2 infection) the use of pressurized suspensions with spacer chamber is suggested. Conversely, the use of nebulisers is not recommended in order to avoid particles aerosolization and increased contagiousness.

Ongoing steroid treatment should not be stopped [21].

**Venous thromboembolism prophylaxis:** severe COVID-19 seems to be associated in adults with an increased risk of disseminated intravascular coagulation and venous thromboembolism. A study on 449 adult patients with severe infection showed a lower mortality rate in those receiving anticoagulant therapy [22]. Therefore, recently adults protocols suggest strategies for prevention and management of coagulative disorders secondary to COVID19 infection with low molecular-weight heparin, especially in case of ARDS [13, 23]. However, children have a much lower incidence of thrombotic complications than adults, even under higher risk conditions such as major surgery or polytrauma [24]*.* Therefore, such prophylaxis is not routinely suggested in children.

An exception can be made for neonatal age and adolescents, where constitutionally the incidence of thrombotic complications is higher [25]. Preventive anticoagulant therapy can therefore be considered for these age groups, in cases where severe inflammatory conditions occur and therefore hyperactivation of the clotting process could lead to the appearance of important thrombotic complications.

The suggested treatment is with subcutaneous enoxaparin 100–200 U/kg/day, that can be increased to 150–300 U/kg/day in neonates.

#### Antiviral treatment

##### Lopinavir/ritonavir

Lopinavir/ritonavir is a boosted protease inhibitor, used, in association with other drugs, in the therapy of *Human Immunodeficiency Virus* (HIV) infection, starting from the age of 14 days of live [26].

Its security profile is well known, as it has been largely used in HIV infections in the pediatric age,

It is available both in oral suspension and tablets.

On the base of some clinical trials, the Chinese guideline on SARS-Cov-2 pneumoniae recommend the early use of lopinavir/ritonavir [27].

On 18 March 2020, the results of a double blinded, randomized, open-label trial, which compared lopinavir/ritonavir associated to standard of care and standard of care alone, on 199 SARS-Cov-2 pneumonia hospitalized patients, have been published on *New England Journal of Medicine* [28]. The study did not evidence any statistically significant differences in the clinical improvement. Mortality at 28 days showed a 5.8% difference in favor of lopinavir/ritonavir use, although not statistically significant (95% Confidence Interval, CI95%: − 17.3-5.7). Patients in which lopinavir/ritonavir therapy has been started before the 12 days of symptoms had a significantly reduction of symptoms duration, hazard ratio: 1.25 (95%CI, 1.77–2.05). Data about mortality, stay in Intensive Care Unit and duration of hospitalization were not stratified for the start of therapy before and after 12 days. The results of this study cannot be transferred to pediatric population, to patients with mild-moderate symptoms, and to patients who underwent an early therapy.

Currently, American guidelines on COVID-19 treatment published in May 2020, recommend both in children and adults to use lopinavir/ritonavir only in the context of clinical trials, given the lack of effectiveness reported now in literature [9, 12]. The latest Chinese guidelines on SARS-Cov-2 pneumoniae do not recommend the use of a specific antiviral for the treatment of COVID-19, and nevertheless include lopinavir/ritonavir among the available therapeutic options for hospitalized patients [29]. Less than 40 clinical trials are currently registered to evaluate the effectiveness of lopinavir/ritonavir against COVID-19.

Lopinavir/ritonavir **is not** indicated in premature neonates before the 42 weeks of corrected age and in all cases before 14 days of live [30].

Available formulations:

Lopinavir/ritonavir tablets (200 mg + 50 mg).

Lopinavir/ritonavir oral suspension (80 mg + 20 mg/mL), which must be conserved in the fridge.

Dosage:

- Adults: 400/100 mg (2 tablets) twice a day
- 14 days − 12 months: 300 mg/75 mg/m^2^ (corresponding to a 3.75 mL/kg) twice a day OR 16/4 mg/kg (corresponding to 0.2 mL/kg) twice a day
- > 12 months - 18 years: if < 15 kg: 12/3 mg/kg (corresponding to 0.15 mL/kg) twice a day; if > 15 kg: 10/2.5 mg/kg (corresponding to 0.125 mL/kg) twice a day

Simplified dosage

- if > 15 kg and able to swallow tablets:

15–25 kg: 1 tablet twice a day.

> 25 kg–35 kg: 1 + ½ tablet twice a day.

> 35 kg: 2 tablets twice a day

- if < 15 kg or unable to swallow tablets:

7–10 kg: 1.25 mL twice a day.

10–15 kg: 1.75 mL twice a day.

15–20 kg: 2.25 mL twice a day.

20–25 kg; 2.75 mL twice a day.

25–30 kg: 3.5 mL twice a day.

30–35 kg: 4 mL twice a day.

35–40 kg: 4.75 mL twice a day.

> 40 kg: 5 mL twice a day.

##### Remdesivir

Remdesivir is a nucleotide analogue, which is incorporated in the viral RNA chain, determining its premature termination. It has been developed from Gilead in 2017 for Ebola therapy. In vitro studies, its high spectrum efficacy against different coronavirus has been demonstrated [31, 32].

Interestingly, remdesivir seems to have a high genetic barrier to viral resistance development (demonstrated in SARS studies). It has a short plasmatic half-life, but it is rapidly converted into its active form (in 2 h from the infusion) and it has a 14 h intracellular half-life [33].

In May 2020, following an assessment of the emergency use authorization criteria and available scientific evidence, the FDA issued an emergency use authorization allowing for the administration of remdesivir intravenously by health care providers for the treatment of COVID-19 suspected or laboratory-confirmed in adults and pediatric patients hospitalized with severe disease [34].

In a guidance approved by the American Pediatric Infectious Diseases Society for COVID-19 treatment in children, if an antiviral is used, panel suggested remdesivir as the preferred agent [12]. Antivirals should preferably be used as part of a clinical trial, if available.

However, a warning has been issued on the co-administration of remdesivir and chloroquine phosphate or hydroxychloroquine sulfate as it may result in reduced antiviral activity of remdesivir [34].

As at 26 June 2020, there are 39 ongoing trials evaluating clinical efficacy of this drug in the therapy of moderate and severe SARS-Cov-2 infections [35]. Four studies (GS-US-540-5774, GS-US-540-5773, GS-US-540-5821 and GS-US-540-5823) are currently ongoing in Italy. All these studies are sponsored by Gilead and only one study involve children from birth to 18 years whereas the other three enroll children older than 12 years and adults [36].

Dosage:

- Adults: 1st day 200 mg IV in 30 min, followed by 100 mg IV /day for other 9 days
- Children (< 40 kg): 1st day 5 mg/kg IV (in 30 min), followed by 2.5 mg/kg IV (in 30 min)/day for other 9 days.

At present, the dosage has not been established for the first 2 weeks of life and weight < 2.5 kg.

##### Hydroxychloroquine

Hydroxychloroquine (and chloroquine) could have an antiviral activity [37]. The mechanism is currently not fully clarified. However, in vitro studies suggest that they could act by increasing the endosomal pH required for virus / host cell fusion and interfering with the glycosylation of the SARS-Cov-2 cell receptor [38]. In particular, hydroxychloroquine appears to have better in vitro activity towards SARS-CoV-2. The anti-inflammatory activity of these molecules, through the inhibition of the production of Interleukin (IL) -6 and Tumor Necrosis Factor (TNF)-α, could contribute to their effectiveness. In addition, these molecules have been in use for decades, showing a good safety profile. Compared to chloroquine, hydroxychloroquine is a drug more readily available and with a higher safety profile [39].

In February 2020, a panel of experts in China summarized the results of the use of chloroquine (500 mg every 12 h for 10 days) in adults with COVID-19, suggesting that its use would be associated with an improvement in the clinical success rate, reducing the hospitalization and improving the outcome [40].

Since then, hydroxychloroquine has been widely used in adult patients [41, 42]. To date, there are more than 200 registered clinical trials for pre-exposure or post-exposure prophylaxis of SARS-CoV-2 infection, and treatment of patients with mild, moderate, and severe COVID-19 [35].

However, it is not yet clear if the benefits outweigh the risks, especially in children. In fact, on 24 April 2020, the FDA warned against the use of hydroxychloroquine or chloroquine for COVID-19 outside hospital or clinical trial setting due to the risk of QT interval prolongation and ventricular tachycardia [43]. This risk could be also increased when combined with some antibiotics such as azithromycin.

Moreover, since the majority of children manifest only mild symptoms, a widespread use of hydroxychloroquine may confer only minimal benefit. Furthermore, pharmacokinetic modeling and simulation used to identify pediatric-specific dosages for hydroxychloroquine have raised concerns on plasma exposures lower than those needed to mediate an antiviral effect [44]. However, these findings do not exclude a potential utility of hydroxychloroquine for COVID-19 treatment based on different mechanisms of action, such as immunomodulation. Conclusive data are needed from ongoing trial to understand the possible role of this drug in children with COVID-19.

Available formulations:

Hydroxychloroquine tablets 200 mg.

Dosage:

Adults: 400 mg twice a day the first day, followed by 200 mg twice a day for overall 5–7 days.

Children: 6 mg/kg (maximum: 400 mg/dose) twice a day on day 1, followed by 3 mg/kg (maximum: 200 mg/dose) twice a day for up to 5 days. *.

* hydroxychloroquine solution preparation is recommended for children.

Precautions for use:

Perform ECG before administering the drug to rule out long QT.

In case of risk factors, dose G6PDH before use.

##### Other antiviral therapy

Favipiravir is an antiviral drug authorized in Japan for the treatment of flu. It works by inhibiting RNA polymerase-RNA-dependent. The medicine is not authorized in Europe or in the USA.

Preliminary results on 80 patients with non-serious SARS-Cov-2 infection, published only in Chinese, seems to show a better efficacy of this drug compared to lopinavir/ritonavir [37].

Few clinical trials are currently undergoing to evaluate the efficacy of Favipiravir in SARS-Cov-2 infection and the AIFA Scientific Technical Commission approved a clinical trial program for this drug [36, 45]*.*

Ivermectin, an FDA-approved anti-parasitic, seems to have broad-spectrum anti-viral activity in vitro, is an inhibitor of the causative virus (SARS-CoV-2), 2 h post infection with SARS-CoV-2 able to effect 5000-fold reduction in viral RNA at 48 h [46]. A single center, double-blind, randomized, placebo-controlled trial is ongoing on ivermectin efficacy in reducing SARS-CoV-2 viral load in adult patients with low risk, non-severe COVID-19 [47].

#### Immunomodulant treatment

##### Steroids

At present, there are no clear evidences to support the use of systemic steroids during SARS-Cov-2 infection unless specific needs (e.g. asthmatic patient who does not respond to 3 doses of bronchodilator, severe allergic reaction). In particular, in patients on chronic systemic or inhaled steroid therapy, treatment stopping is not necessary. Some data suggest that methylprednisolone could have an immunomodulating activity in case of ARDS, decreasing the risk of death [48]. Its use is also indicated in case of a worsening of pulmonary function after at least 7 days from the beginning of symptoms, in association with marked alteration or tendency to increasing of IL- 6 and/or D-dimer and/or ferritin and/or C-reactive protein.

In these cases, can be used:

- Methylprednisolone 1–2 mg / kg (max 80 mg) once a day.

A short course is indicated (2–5 days).

In severe cases high doses (methylprednisolone 30 mg/kg) can be considered.

Recently some good results were reported by the interim analysis of the RECOVERY trial [49], a study where 2104 adult patients were randomised to receive dexamethasone 6 mg once per day (either orally or intravenously) for 10 days and 4321 patients were randomised to usual care alone. Dexamethasone significantly reduced deaths by one-third in ventilated patients (relative risk, RR 0.65) and by one fifth in those receiving only oxygen (RR 0.80), but there was no benefit among patients who did not require respiratory support. Based on these results, a treatment with dexamethasone (0.2–0.4 mg/kg, maximum 6 mg) in patients requiring oxygen should be considered.

##### Anakinra

The multisystem inflammatory syndrome reported in patients with COVID-19 shares considerable biochemical overlapping with the cytokine storm complicating macrophage activation syndrome associated with rheumatic disease. Anakinra is licensed for treating people with rheumatoid arthritis, aged at least 8 months. This is a 17 kD recombinant, non-glycosylated human IL-1 receptor antagonist who inhibits the proinflammatory cytokines interleukin (IL)-1α and IL-1β, with a short half-life of about 3–4 h and good safety profile. Anakinra has also been used with some success to treat macrophage activation syndrome caused by various inflammatory conditions.

Several case series report that Anakinra reduced both need for invasive mechanical ventilation in the intensive care unit and mortality among patients with severe forms of COVID-19, without serious side-effects [50, 51]. Currently, there are 10 ongoing clinical trials on Anakinra use in COVID-19, all in adults [52]. However, there are few pediatric reports of children treated with Anakinra showing that such treatment in serious cases can be safe and beneficial [53, 54].

Indeed, anakinra has been proposed as the best option if such treatment is considered necessary, because it has a relatively short half-life and may be discontinued rapidly in case of adverse effect or concern of worsening the infection [55]. Anakinra can be administered intravenously (off label) or subcutaneously and has a wide therapeutic window; when anakinra is effective for cytokine storm syndromes, it works within 2 to 3 days [56].

Therapeutic scheme:

- Anakinra vial 100 mg/0.67 ml
- intravenously: 8–10 mg/kg/day in 2 or 4 administrations depending on the total dose (up to a maximum of 100 mg 4 times a day)

After 48–72 h, repeat the plasma dosage of IL-6 and/or D-dimer.

##### Tocilizumab

Tocilizumab is a recombinant humanized monoclonal antibody belonging to the G1 immunoglobulin subclass and directed against both soluble and membrane IL-6 receptors [57].

This drug is indicated for the treatment of moderate and severe rheumatoid arthritis, systemic juvenile idiopathic arthritis (from the age of 1 year), juvenile idiopathic polyarthritis (from the age of 2 years) and severe release of cytokines induced by CAR-T lymphocytes (chimeric antigen receptor t cell) (from the age of 2 years).

Studies suggested that the alveolar damage in COVID-19 is caused by a cytokine storm (including IL-6) and that symptoms improve with the use of Tocilizumab [58, 59].

Based on these results, different clinical trials have been started. The Italian multicenter study TOCIVID-19 has been promoted by the National Cancer Institute, IRCCS, G. Pascale Foundation, Naples. The recruitment of the prospective phase has been completed, while the observational study continues. The protocol includes the enrollment of patients of any age.

Two other studies started at the end of March in Italy, but not enrolling pediatric patients. One of them, aimed at assessing the efficacy of the early tocilizumab administered early in patients suffering from recently emerging Covid-19 pneumonia and requiring hospital care but not invasive or semi-invasive ventilation, was concluded early because it was not effective [36].

Therapeutic scheme:

- Tocilizumab vial 20 mg/mL
- First infusion at a dosage of 10–12 mg/kg < 30 kg and 8 mg/kg > 30 kg, (maximum dosage 800 mg, duration of infusion at least 60 min)
- Second infusion 12 h after the first (at the discretion of the doctor, in case of no response)
- A possible third infusion after 24 h may be considered.

After 24 h from the last administration, repeat the plasma dosage of IL-6 and / or D-dimer.

When to use biologic drugs:

• Serious or critical cases.

• End of the initial phase of high viral load of COVID-19 (afebrile> 72 h and/or at least 7 days after the onset of symptoms).

• High levels of IL-6 (> 40 pg/ml); alternatively, high levels of d-dimer and/or PCR and/or ferritin and/or fibrinogen increasing progressively.

When not to use biologic drugs:

• AST / ALT value above 5 times normal levels.

• Neutrophils value lower than 500 cells/mL.

• Platelets value lower than 50,000 cells /mL.

• Documented sepsis from other pathogens other than COVID-19.

• Presence of comorbidities related, according to clinical judgment, to an unfavorable outcome.

• Complicated diverticulitis or intestinal perforation.

• Immunomodulating and anti-rejection therapy.

• Known hypersensitivity to the drug.

It is also recommended to avoid administration of biologics if anti-MRPV vaccination has been carried out in the past 30 days.

If possible, before starting therapy:

- Quantiferon test
- HBV and HCV markers

##### Other immunomodulant therapies

In view of the fact that the alveolar damage of severe forms of SARS-Cov-2 infection is caused by a cytokine storm [60], two other clinical trials have been started in Italy on the use of emapalumab (anti-interferon gamma monoclonal antibody) and associated anakinra (IL-1 receptor antagonist) and the use of sarilumab (monoclonal antibody that binds to the IL-6 receptor). A Belgian study comparing tocilizumab, anakinra and siltuximab is going to start soon.

Information and forms relating to the studies are available on AIFA website, but there is no prevision for enrollment of pediatric patients [36].

Among the new therapies planned for the future is the infusion of hyperimmune plasma from cured patients. This approach, already used in China and previously for Ebola and SARS, seems to give good results at least in the most serious cases. In the USA 3 studies have been launched that will evaluate this approach [61].

#### Antibiotic therapy

The choice to add empirical antibiotic therapy should only be made if there is reasonable evidence of bacterial superinfection. An increased procalcitonin, C-reactive protein and the presence of neutrophilic leukocytosis represent laboratory parameters suggestive of bacterial infection. From a clinical point of view, the persistence of fever for more than 3 days can be suggestive of bacterial infection [62]. Tests for atypical bacteria (i.e. mycoplasma) should be performed in case of clinical suspect, in order to start targeted therapy.

The start of an empirical antibiotic therapy is also recommended in the presence of comorbidities, such as immunodeficiency, cystic fibrosis, other chronic diseases of the respiratory tract, severe neuromotor disability.

In case of productive cough, the collection of a sputum sample for culture examination before the start of antibiotic therapy would be indicated.

In patients without risk factors, we recommend:

- Amoxicillin 90 mg /kg/day in 3 doses, in case of possible oral intake
- Ceftriaxone 80–100 mg/kg/day, in case of impossibility of oral intake. This drug is also recommended for the possibility of administering once a day and therefore reducing the risks for healthcare professionals.

##### Azithromycin

Preliminary data have suggested a possible efficacy of azithromycin in combination with hydroxychloroquine as a background therapy for SARS-Cov-2 infection. The exact role of this drug in COVID-19 is unknown, as no in vitro data are available at present. It has been supposed a dual role, antiviral and anti-inflammatory. The anti-inflammatory action of azithromycin has been already demonstrated in many conditions. Regarding the theoretical antiviral activity, the first published study was on a small sample size (36 patients, of whom only 6 receiving the combination of azithromycin/hydroxychloroquine) [41]. In this study, by Gautret and colleagues, 100% of the patients taking hydroxychloroquine and azithromycin had negative nasopharyngeal swab 6 days from the start of therapy, compared to 57.1% of the hydroxychloroquine alone and 12.5% ​​of the control group. The same results have been replicated in a larger population [63]. However, a more recent study on virological and clinical results of 11 adult patients with a serious disease raises doubts about the antiviral efficacy of this combination, as it reports results in contrast with those of Gautret et al. [64].

This combination have been also evaluated in a larger observational study including 1061 adults, resulting to be safe and associate with a very low fatality rate. However, its efficacy has been doubted in a study on a large adult population in New York among hospitalized patients with COVID-19, where treatment with hydroxychloroquine, azithromycin, or both were not associated with significantly lower in-hospital mortality [65]. Considering the lack of a strong rationale and the absence of evidence of certain effectiveness in the treatment of COVID-19 patients, the use of azithromycin should be considered carefully and the QTc interval strongly monitored. In fact, on April 2020, European Medicines Agency (EMA) warned about risk of serious side effects particularly if associated with chloroquine and hydroxychloroquine [66].

Dosage:

- Adults: 500 mg the first day, then 250 mg/day for other 4 days
- Children: 15 mg/kg the first day, then 7.5 mg/kg once a day for other 4 days

### Drug interactions

The drugs used for the therapy of SARS-Cov-2 infection, especially lopinavir/ritonavir and azithromycin/ hydroxychloroquine, can have interactions with other drugs.

Before administering those treatment, evaluate possible interactions on drugs [67].

### Informed consent

For all drugs, consent for off-label use must be requested and the procedures provided by the reference Healthcare Company must be followed.

As for Remdesivir, since it is an experimental drug, the procedures for compassionate use must be followed.

## Conclusion

In conclusion this position paper summarizes the suggested treatments in COVID-19 infected children based on a review of the current literature carried out by the Scientific Committee of the Italian Society of Infectious Pediatric Diseases. Since most SARS-CoV-2 infections in children have a benign course, pharmacological treatment, other than supportive therapy, should be reserved to those with more severe cases.

As new evidences are expected to be available in the next weeks/months, the Italian Society of Pediatric Infectious Diseases will guarantee to maintain treatment indication updated on the website www.sitip.org

## Data Availability

All the publication are listed in bibliography.

## Abbreviations

AIFA: Agenzia Italiana del Farmaco
AST / ALT: Aspartate & Alkaline transaminase
COVID-19: Coronavirus 2019
ECG: electrocardiogram
FDA: Food and Drug Administration
HIV: Human Immunodeficiency Virus
PARDS: Pediatric acute respiratory distress syndrome
SARS-CoV-2: severe acute respiratory syndrome coronavirus 2
SpO2: Oxygen saturation
TNF: Tumor Necrosis Factor
WHO: World Health Organization

## References

[1] World Health Organization. WHO Director-General's opening remarks at the media briefing on COVID-19 - 11 March 2020. https://www.who.int/dg/speeches/detail/who-director-general-s-opening-remarks-at-the-media-briefing-on-covid-19%2D%2D-11-march-2020. Accessed 16 Jun 2020.

[2] Ludvigsson JF. Systematic review of COVID-19 in children shows milder cases and a better prognosis than adults. Acta Paediatr. 2020. 10.1111/apa.15270. 10.1111/apa.15270 PMC7228328 32202343

[3] Garazzino S, Montagnani C, Donà D, Meini A, Felici E, Vergine G, the Italian SITIP-SIP Pediatric Infection Study Group (2020). Multicentre Italian study of SARS-CoV-2 infection in children and adolescents, preliminary data as at 10 April 2020. Euro Surveill.

[4] Tagarro A, Epalza C, Santos M, Sanz-Santaeufemia FJ, Otheo E, Moraleda C, et al. Screening and severity of coronavirus disease 2019 (COVID-19) in children in Madrid, Spain. JAMA Pediatr. 2020. 10.1001/jamapediatrics.2020.1346. 10.1001/jamapediatrics.2020.1346 PMC7142799 32267485

[5] Sun D, Li H, Lu XX, Xiao H, Ren J, Zhang FR, Liu ZS. Clinical features of severe pediatric patients with coronavirus disease 2019 in Wuhan: a single center's observational study. World J Pediatr. 2020. 10.1007/s12519-020-00354-4. 10.1007/s12519-020-00354-4 PMC7091225 32193831

[6] Toubiana J, Poirault C, Corsia A, Bajolle F, Fourgeaud J, Angoulvant F (2020). Kawasaki-like multisystem inflammatory syndrome in children during the covid-19 pandemic in Paris, France: prospective observational study. BMJ.

[7] Verdoni L, Mazza A, Gervasoni A, Martelli L, Ruggeri M, Ciuffreda M (2020). An outbreak of severe Kawasaki-like disease at the Italian epicentre of the SARS-CoV-2 epidemic: an observational cohort study. Lancet.

[8] Clinicaltrial.gov. Available at: https://clinicaltrials.gov/ct2/results?cond=Covid-19&term=treatment&cntry=&state=&city=&dist=&age=0. Accessed 16 Jun 2020.

[9] National Institute of Health. Coronavirus Disease 2019 (COVID-19) Treatment Guidelines. Available at: https://files.covid19treatmentguidelines.nih.gov/guidelines/covid19treatmentguide lines.pdf Accessed 14 Jun 2020. 34003615

[10] Società Italiana di Malattie Infettive Tropicali. Sezione Regione Lazio. Raccomandazioni per la gestione clinica e terapeutica della COVID-19, 5 Maggio 2020. https://www.simit.org/images/documenti/Linee%20guida%20SIMIT%20LAZIO%20SARS%20CoV%202%20maggio%202020.pdf Accessed 26 Jun 2020.

[11] European Respiratory Society. Available at: https://www.ersnet.org/covid-19-guidelines-and-recommendations-directory. Accessed 16 Jun 2020.

[12] Chiotos K, Hayes M, Kimberlin DW, Jones SB, James SH, Pinninti SG, et al. Multicenter initial guidance on use of antivirals for children with COVID-19/SARS-CoV-2. J Pediatric Infect Dis Soc. 2020;piaa045. 10.1093/jpids/piaa045. 10.1093/jpids/piaa045 PMC7188128 32318706

[13] World Health Organization. Clinical management of severe acute respiratory infection when novel coronavirus (nCoV) infection is suspected: Interim Guidance 13 March 2020. https://www.who.int/publications-detail/clinical-management-of-severe-acute-respiratory-infection-when-novel-coronavirus-(ncov)-infection-is-suspected. Accessed 26 Jun 2020.

[14] World Health Organization. Multisystem inflammatory syndrome in children and adolescents with COVID-19. Available at: https://www.who.int/publications/i/item/multisystem-inflammatory-syndrome-in-children-and-adolescents-with-covid-19. Accessed 16 Jun 2020.

[15] World Health Organization. Available at: https://www.who.int/news-room/commentaries/detail/multisystem-inflammatory-syndrome-in-children-and-adolescents-with-covid-19. Accessed 26 Jun 2020.

[16] Pediatric Acute Lung Injury Consensus Conference Group (2015). Pediatric acute respiratory distress syndrome: consensus recommendations from the pediatric acute lung injury consensus conference. Pediatr Crit Care Med.

[17] Weiss SL, Peters MJ, Alhazzani W, Agus MSD, Flori HR, Inwald DP (2020). Surviving Sepsis campaign international guidelines for the Management of Septic Shock and Sepsis-Associated Organ Dysfunction in children. Pediatr Crit Care Med.

[18] Parshuram CS, Duncan HP, Joffe AR, Farrell CA, Lacroix JR, Middaugh KL (2011). Multicentre validation of the bedside paediatric early warning system score: a severity of illness score to detect evolving critical illness in hospitalised children. Crit Care.

[19] Day M (2020). Covid-19: ibuprofen should not be used for managing symptoms, say doctors and scientists. BMJ..

[20] European Medicines Agency. EMA gives advice on the use of non-steroidal anti-inflammatories for COVID-19. March 2020. https://www.ema.europa.eu/en/news/ema-gives-advice-use-non-steroidal-anti-inflammatories-covid-19. Accessed 16 Jun 2020.

[21] Global Initiative for Asthma (GINA). Recommendations for inhaled asthma controller medications. March 19, 2020. https://ginasthma.org/recommendations-for-inhaled-asthma-controller-medications/. Accessed 14 Jun 2020.

[22] Tang N, Bai H, Chen X, Gong J, Li D, Sun Z. Anticoagulant treatment is associated with decreased mortality in severe coronavirus disease 2019 patients with coagulopathy. J Thromb Haemost. 2020 Mar 27. 10.1111/jth.14817 [Epub ahead of print]. 10.1111/jth.14817 PMC9906401 32220112

[23] Tachil J, Tang N, Gando S, Falanga A, Cattaneo M, Levi M, Clark C, Iba T. ISTH interim guidance on recognition and management of coagulopathy in COVID-19. J Thromb Haemost. 2020; [Epub ahead of print]. 10.1111/jth.14810 PMC9906133 32338827

[24] Mahajerin A, Branchford BR, Amankwah EK, Raffini L, Chalmers E, van Ommen CH, Goldenberg NA (2015). Hospital-associated venous thromboembolism in pediatrics: a systematic review and meta-analysis of risk factors and risk-assessment models. Haematologica.

[25] Jaffray J, Young G (2013). Developmental hemostasis: clinical implications from the fetus to the adolescent. Pediatr Clin N Am.

[26] Bamford A, Turkova A, Lyall H, Foster C, Klein N, Bastiaans D (2018). Paediatric European Network for Treatment of AIDS (PENTA) guidelines for treatment of paediatric HIV-1 infection 2015: optimizing health in preparation for adult life. HIV Med.

[27] National Health Commission & State Administration of Traditional Chinese Medicine. Diagnosis and Treatment Protocol for Novel Coronavirus Pneumonia. March 3, 2020. http://www.kankyokansen.org/uploads/uploads/files/jsipc/protocol_V7.pdf. Accessed 16 Jun 2020. 10.1097/IM9.0000000000000022 PMC7227203 40479241

[28] Cao B, Wang Y, Wen D, Liu W, Wang J, Fan G, et al. A trial of Lopinavir-ritonavir in adults hospitalized with severe Covid-19. N Engl J Med. 2020. 10.1056/NEJMoa2001282. 10.1056/NEJMoa2001282 PMC7121492 32187464

[29] National Health Commission & National Administration of Traditional Chinese Medicine (2020). Diagnosis and treatment protocol for novel coronavirus pneumonia (trial version 7). Chin Med J.

[30] European Medicines Agency. European public assessment report (EPAR) for Kaletra. https://www.ema.europa.eu/en/medicines/human/EPAR/kaletra. Accessed 16 Jun 2020.

[31] Ko WC, Rolain JM, Lee NY, Chen PL, Huang CT, Lee PI, et al. Arguments in favour of remdesivir for treating SARS-CoV-2 infections. Int J Antimicrob Agents. 2020. 10.1016/j.ijantimicag.2020.105933. 10.1016/j.ijantimicag.2020.105933 PMC7135364 32147516

[32] Al-Tawfiq JA, Al-Homoud AH, Memish ZA. Remdesivir as a possible therapeutic option for the COVID-19. Travel Med Infect Dis. 2020. 10.1016/j.tmaid.2020.101615. 10.1016/j.tmaid.2020.101615 PMC7129391 32145386

[33] Wang M, Cao R, Zhang L, Yang X, Liu J, Xu M (2020). Remdesivir and chloroquine effectively inhibit the recently emerged novel coronavirus (2019-nCoV) in vitro. Cell Res.

[34] Food and Drug Administration. Remdesivir by Gilead Sciences: FDA Warns of Newly Discovered Potential Drug Interaction That May Reduce Effectiveness of Treatment. Available at: https://www.fda.gov/safety/medical-product-safety-information/remdesivir-gilead-sciences-fda-warns-newly-discovered-potential-drug-interaction-may-reduce. Accessed 14 Jun 2020.

[35] Clinicaltrials.gov. Available at: https://clinicaltrials.gov/ct2/results?cond=covid-19&term=hydroxychloroquine&cntry=&state=&city=&dist= Accessed 16 Jun 2020.

[36] Sperimentazioni cliniche - COVID-19. https://www.aifa.gov.it/sperimentazioni-cliniche-covid-19. Accessed 26 Jun 2020.

[37] Dong L, Hu S, Gao J (2020). Discovering drugs to treat coronavirus disease 2019 (COVID-19). Drug Discov Ther.

[38] Gao J, Tian Z, Yang X (2020). Breakthrough: Chloroquine phosphate has shown apparent efficacy in treatment of COVID-19 associated pneumonia in clinical studies. Biosci Trends.

[39] Yao X, Ye F, Zhang M, Cui C, Huang B, Niu P, et al. In Vitro Antiviral Activity and Projection of Optimized Dosing Design of Hydroxychloroquine for the Treatment of Severe Acute Respiratory Syndrome Coronavirus 2 (SARS-CoV-2). Clin Infect Dis. 2020. 10.1093/cid/ciaa237. 10.1093/cid/ciaa237 PMC7108130 32150618

[40] Zhonghua Jie, He He, Hu Xi, Za Zhi for the multicenter collaboration group of Department of Science and Technology of Guangdong Province and Health Commission of Guangdong Province for chloroquine in the treatment of novel coronavirus pneumonia. Expert consensus on chloroquine phosphate for the treatment of novel coronavirus pneumonia (Article in Chinese) 2020 Mar 12;43(3):185–188. doi: 10.3760/cma.j.issn.1001-0939.2020.03.009. 10.3760/cma.j.issn.1001-0939.2020.03.009 32164085

[41] Gautret P, Lagier JC, Parola P, Hoang VT, Meddeb L, Mailhe M, et al. Hydroxychloroquine and azithromycin as a treatment of COVID-19: results of an open-label non-randomized clinical trial. Int J Antimicrob Agents. 2020. 10.1016/j.ijantimicag.2020.105949. 10.1016/j.ijantimicag.2020.105949 PMC7102549 32205204

[42] Centers for Disease Control and Prevention. Information for Clinicians on Therapeutic Options for COVID-19 Patients. March 21, 2020. Available at: https://www.cdc.gov/coronavirus/2019-ncov/hcp/therapeutic-options.html. Last accessed: 27 Mar 2020.

[43] Food and Drug Administration. FDA cautions against use of hydroxychloroquine or chloroquine for COVID-19 outside of the hospital setting or a clinical trial due to risk of heart rhythm problems. Available at: https://www.fda.gov/drugs/fda-drug-safety-podcasts/fda-cautions-against-use-hydroxychloroquine-or-chloroquine-covid-19-outside-hospital-setting-or. Accessed 14 Jun 2020.

[44] Maharaj AR, Wu H, Hornik CP, Balevic SJ, Hornik CD, Smith PB, Best Pharmaceuticals for Children Act–Pediatric Trials Network Steering Committee, et al. Simulated Assessment of Pharmacokinetically Guided Dosing for Investigational Treatments of Pediatric Patients With Coronavirus Disease 2019. JAMA Pediatr:2020. 10.1001/jamapediatrics.2020.2422. 10.1001/jamapediatrics.2020.2422 PMC7275264 32501511

[45] Favipiravir: aggiornamento della valutazione della CTS. https://www.aifa.gov.it/-/covid-19-autorizzato-nuovo-studio-clinico-con-favipiravir. Accessed 24 Jun 2020.

[46] Caly L, Druce JD, Catton MG, Jans DA, Wagstaff KM. The FDA-approved drug Ivermectin inhibits the replication of SARS-CoV-2 in vitro. Antivir Res. 2020;104787 [Epub ahead of print]. 10.1016/j.antiviral.2020.104787 PMC7129059 32251768

[47] Chaccour C, Ruiz-Castillo P, Richardson MA, Moncunill G, Casellas A, Carmona-Torre F (2020). The SARS-CoV-2 Ivermectin Navarra-ISGlobal Trial (SAINT) to Evaluate the Potential of Ivermectin to Reduce COVID-19 Transmission in low risk, non-severe COVID-19 patients in the first 48 hours after symptoms onset: A structured summary of a study protocol for a randomized control pilot trial. Trials.

[48] Wu C, Chen X, Cai Y, Xia J, Zhou X, Xu S, et al. Risk factors associated with acute respiratory distress syndrome and death in patients with coronavirus disease 2019 pneumonia in Wuhan, China. JAMA Intern Med. 2020. 10.1001/jamainternmed.2020.0994. 10.1001/jamainternmed.2020.0994 PMC7070509 32167524

[49] ^R^ECOVERY trial. Available at: https://www.recoverytrial.net/news/low-cost-dexamethasone-reduces-death-by-up-to-one-third-in-hospitalised-patients-with-severe-respiratory-complications-of-covid-19. Accessed 16 Jun 2020.

[50] Huet T, Beaussier H, Voisin O, Jouveshomme S, Dauriat G, Lazareth I, et al. Anakinra for severe forms of COVID-19: a cohort study. Lancet Rheumatol. 2020. 10.1016/S2665-9913(20)30164-8. 10.1016/S2665-9913(20)30164-8 PMC7259909 32835245

[51] Cavalli G, De Luca G, Campochiaro C, Della-Torre E, Ripa M, Canetti D, et al. Interleukin-1 blockade with high-dose anakinra in patients with COVID-19, acute respiratory distress syndrome, and hyperinflammation: a retrospective cohort study. Lancet Rheumatol. 2020. 10.1016/S2665-9913(20)30127-2. 10.1016/S2665-9913(20)30127-2 PMC7252085 32501454

[52] King A, Vail A, O'Leary C, Hannan C, Brough D, Patel H (2020). Anakinra in COVID-19: important considerations for clinical trials the lancet Reumatology.

[53] Whittaker E, Bamford A, Kenny J, Kaforou M, Jones CE, Shah P, et al. Clinical Characteristics of 58 Children With a Pediatric Inflammatory Multisystem Syndrome Temporally Associated With SARS-CoV-2. JAMA. 2020. 10.1001/jama.2020.10369. 10.1001/jama.2020.10369 PMC7281356 32511692

[54] Pain EC, Felsenstein S, Cleary G, Mayell S, Conrad K, Harave S, et al. Novel paediatric presentation of COVID-19 with ARDS and cytokine storm syndrome without respiratory symptoms. Lancet Rheumatol. 2020. 10.1016/S2665-9913(20)30137-5. 10.1016/S2665-9913(20)30137-5 PMC7228732 32427161

[55] Wampler Muskardin TL (2020). Intravenous Anakinra for macrophage activation syndrome may hold lessons for treatment of cytokine storm in the setting of coronavirus disease 2019. ACR.

[56] Cron R, Chatham W (2020). The Rheumatologist’s role in COVID-19. J Rheumatol.

[57] European Medicines Agency. European public assessment report (EPAR) for RoActemra (tocilizumab). https://www.ema.europa.eu/en/medicines/human/EPAR/roactemra. Accessed 4 Apr 2020.

[58] European Medicines Agency. EMA gives advice on the use of non-steroidal anti- inflammatories for COVID-19. March 18, 2020. Available at:https://www.ema.europa.eu/en/documents/press-release/ema-gives-advice-use-non-steroidal-anti-inflammatories-covid-19_en.pdf. Accessed 14 Jun 2020.

[59] Xu X, Han M, Li T, Sun W , Wang D , Fu B, et al. Effective treatment of severe COVID-19 patients with Tocilizumab. chinaXiv:202003.00026v1. Accessed 4 Apr 2020. 10.1073/pnas.2005615117 PMC7245089 32350134

[60] Mehta P, McAuley DF, Brown M (2020). COVID-19: consider cytokine storm syndromes and immunosuppression. Lancet.

[61] Maxmen A (2020). How blood from coronavirus survivors might save lives. Nature.

[62] Chen ZM, Fu JF, Shu Q, Chen YH, Hua CZ, Li FB, et al. Diagnosis and treatment recommendations for pediatric respiratory infection caused by the 2019 novel coronavirus. World J Pediatr. 2020. 10.1007/s12519-020-00345-5. 10.1007/s12519-020-00345-5 PMC7091166 32026148

[63] Gautret P, Lagier JC, Parola P, Hoang VT, Meddeb L, Sevestre J (2020). Clinical and microbiological effect of a combination of hydroxychloroquine and azithromycin in 80 COVID-19 patients with at least a six-day follow up: A pilot observational study. Travel Med Infect Dis.

[64] Molina JM, Delaugerre C, Le Goff J, Mela-Lima B, Ponscarme D Goldwirt, de Castro N. No Evidence of Rapid Antiviral Clearance or Clinical Benefit with the Combination of Hydroxy-chloroquine and Azithromycin in Patients with Severe COVID-19 Infection. Med Mal Infect 2020 Mar 30 Med Mal Infect [Online ahead of print] - doi: 10.1016/j.medmal.2020.03.006. 10.1016/j.medmal.2020.03.006 PMC7195369 32240719

[65] Rosenberg ES, Dufort EM, Udo T, Wilberschied LA, Kumar J, Tesoriero J, et al. Association of Treatment with Hydroxychloroquine or azithromycin with in-hospital mortality in patients with COVID-19 in New York state. JAMA. 2020;208630. 10.1001/jama.2020.8630. 10.1001/jama.2020.8630 PMC7215635 32392282

[66] European Medicines Agency. COVID-19: reminder of risk of serious side effects with chloroquine and hydroxychloroquine. Available at: https://www.ema.europa.eu/en/news/covid-19-reminder-risk-serious-side-effects-chloroquine-hydroxychloroquine. Accessed 16 Jun 2020.

[67] COVID-19 drug interactions. https://www.covid19-druginteractions.org/. Accessed 4 Apr 2020.

